# The effect of PTC124 on choroideremia fibroblasts and iPSC-derived RPE raises considerations for therapy

**DOI:** 10.1038/s41598-018-26481-7

**Published:** 2018-05-29

**Authors:** Simona Torriano, Nejla Erkilic, David Baux, Nicolas Cereso, Valerie De Luca, Isabelle Meunier, Mariya Moosajee, Anne-Francoise Roux, Christian P. Hamel, Vasiliki Kalatzis

**Affiliations:** 1 Inserm U1051, Institute for Neurosciences of Montpellier, Montpellier, France; 2 University of Montpellier, Montpellier, France; 3 Laboratory of Molecular Genetics, CHRU, Montpellier, France; 4 Department of Ophthalmology, CHRU, Montpellier, France; 5 Centre of Reference for Genetic Sensory Diseases, CHRU, Montpellier, France; 6 UCL, Institute of Ophthalmology, London, UK; 7 Laboratory of Rare Genetic Diseases, EA 7402, University of Montpellier, Montpellier, France

## Abstract

Inherited retinal dystrophies (IRDs) are caused by mutations in over 200 genes, resulting in a range of therapeutic options. Translational read-through inducing drugs (TRIDs) offer the possibility of treating multiple IRDs regardless of the causative gene. TRIDs promote ribosomal misreading of premature stop codons, which results in the incorporation of a near-cognate amino acid to produce a full-length protein. The IRD choroideremia (CHM) is a pertinent candidate for TRID therapy, as nonsense variants cause 30% of cases. Recently, treatment of the UAA nonsense-carrying CHM zebrafish model with the TRID PTC124 corrected the underlying biochemical defect and improved retinal phenotype. To be clinically relevant, we studied PTC124 efficiency in UAA nonsense-carrying human fibroblasts and induced pluripotent stem cell-derived retinal pigment epithelium, as well as in a UAA-mutated *CHM* overexpression system. We showed that PTC124 treatment induces a non-significant trend for functional rescue, which could not be improved by nonsense-mediated decay inhibition. Furthermore, it does not produce a detectable *CHM*-encoded protein even when coupled with a proteasome inhibitor. We suggest that drug efficiency may depend upon on the target amino acid and its evolutionary conservation, and argue that patient cells should be screened *in vitro* prior to inclusion in a clinical trial.

## Introduction

The retina lines the back of the eye and is responsible for capturing, converting and transmitting the light signal to the brain for image interpretation. It is made up of the neuroretina, which contains the light-sensing photoreceptors, and the supporting retinal pigment epithelium (RPE). Inherited retinal dystrophies (IRDs) are a group of clinically heterogeneous disorders that are characterised by degeneration of the photoreceptors and/or RPE. IRDs are also genetically heterogeneous with over 200 causative genes identified to date (https://sph.uth.tmc.edu/Retnet). Collectively, they have a prevalence of ~1 in 2500 individuals^1^. As IRDs are mainly monogenic, they have received much focus as therapeutic targets for gene-dependent approaches, such as viral vector-mediated gene replacement^2–5^.

However, it is unlikely that a successful gene replacement approach will be developed for all IRD genes in the near future. It is thus important to study in parallel other therapeutic options. Mutation-dependent therapies are particularly interesting, as they would reach a larger target population. One option is pharmacological compounds, which, if already approved for human use, could result in rapid clinical translation in the event of positive results. An example is translational read-through inducing drugs (TRIDs). TRIDs work on nonsense mutations by promoting ribosomal misreading of the premature stop codon and resulting in the incorporation of a near-cognate amino acid. This produces a full-length protein, which in some cases can be functional^6^. The most common examples are the widely used class of antibiotics, the aminoglycosides. Although approved for human use, they have the disadvantage of significant oto-^7^ and nephrotoxicity^8^ that precludes long-term use^9^. In addition, retinal toxicity has also been reported following intraocular administration^10,11^.

Studies have been aimed at developing and testing alternative TRIDs with lower side effects^12–14^. One such TRID is PTC124^15^, which has been widely tested and reported to have the efficiency of aminoglycosides without the associated toxicity. Furthermore, this molecule reached phase 3 clinical trial testing for cystic fibrosis (NCT02139306) and Duchenne muscular dystrophy (NCT01826487). In terms of ocular disease, PTC124 has been investigated as a potential TRID for Usher syndrome 1C^16^, retinitis pigmentosa^17,18^ and aniridia^19^. In addition, the treatment of aniridia by PTC124 will be further investigated in a clinical trial (NCT02647359).

The potential interest of TRID therapy increases proportionally with the prevalence of nonsense mutations for a given disease. Along this line, the IRD choroideremia (CHM) is a particularly pertinent candidate as 30% of causative mutations are nonsense variants^20^. Therefore, if successful, this approach would allow the treatment of a non-negligible number of patients. CHM is characterised by a progressive degeneration of the photoreceptors, RPE and underlying choroid. At the clinical level, patients present with night blindness in childhood, and evolve to legal blindness from 40 years of age^21^. CHM is an X-linked disorder due to mutations in the gene *CHM* that encodes Rab escort protein 1 (REP1)^22^. REP1 acts as a chaperon for Rab guanosine triphosphatases, transporting them to the enzyme Rab geranylgeranyl transferase (RGGTase) for prenylation. REP1 then delivers the prenylated Rabs to their target membranes, where they act as regulators of vesicular trafficking^23^. Thus, in the absence of REP1, unprenylated Rabs accumulate in the cytosol.

Interestingly, a zebrafish model of CHM due to a nonsense UAA mutation has been described^24,25^. This model arises from a c.96C > T transition in exon 2 of the *chm* gene that gives rise to the p.Gln33* alteration, which results in lethality from day 5 post-fertilisation (dpf). Nonetheless, the mutant larvae have been exploited as a model for TRID therapy of CHM. It was shown that aminoglycosides (gentamicin and paromomycin) could significantly preserve retinal structure and increase survival of the *chm*^*−/y*^ zebrafish larvae^26^. We more recently showed that PTC124 treatment of *chm*^*−/y*^ larvae, in comparison to gentamicin, corrected the prenylation defect, improved the retinal phenotype and increased survival^27^.

As a clinically pertinent complement to that study, we have tested PTC124 efficiency in human cells carrying a UAA nonsense mutation. We tested the ability of PTC124 to improve the prenylation defect in patient fibroblasts and in RPE derived from patient induced pluripotent stem cells (iPSC). A trend for functional rescue was observed, albeit not significant. *In silico* analysis and 3D modelling suggest that, if read-through is taking place, drug efficiency may depend on the conservation and type of the target amino acid, and its localisation with regards to REP1 structure and Rab binding. However, PTC124 treatment did not result in the expression of detectable REP1 in the patient cells or in an overexpression system comprising CHM:EGFP fusion constructs carrying the UAA mutation. We highlight considerations that should be taken into account when considering TRID therapy for this and other disorders, and suggest that patient cells should be used for drug screening prior to inclusion in a clinical trial.

## Results

### Effect of the c.772A > T variant on *CHM* expression

**Figure 1:**
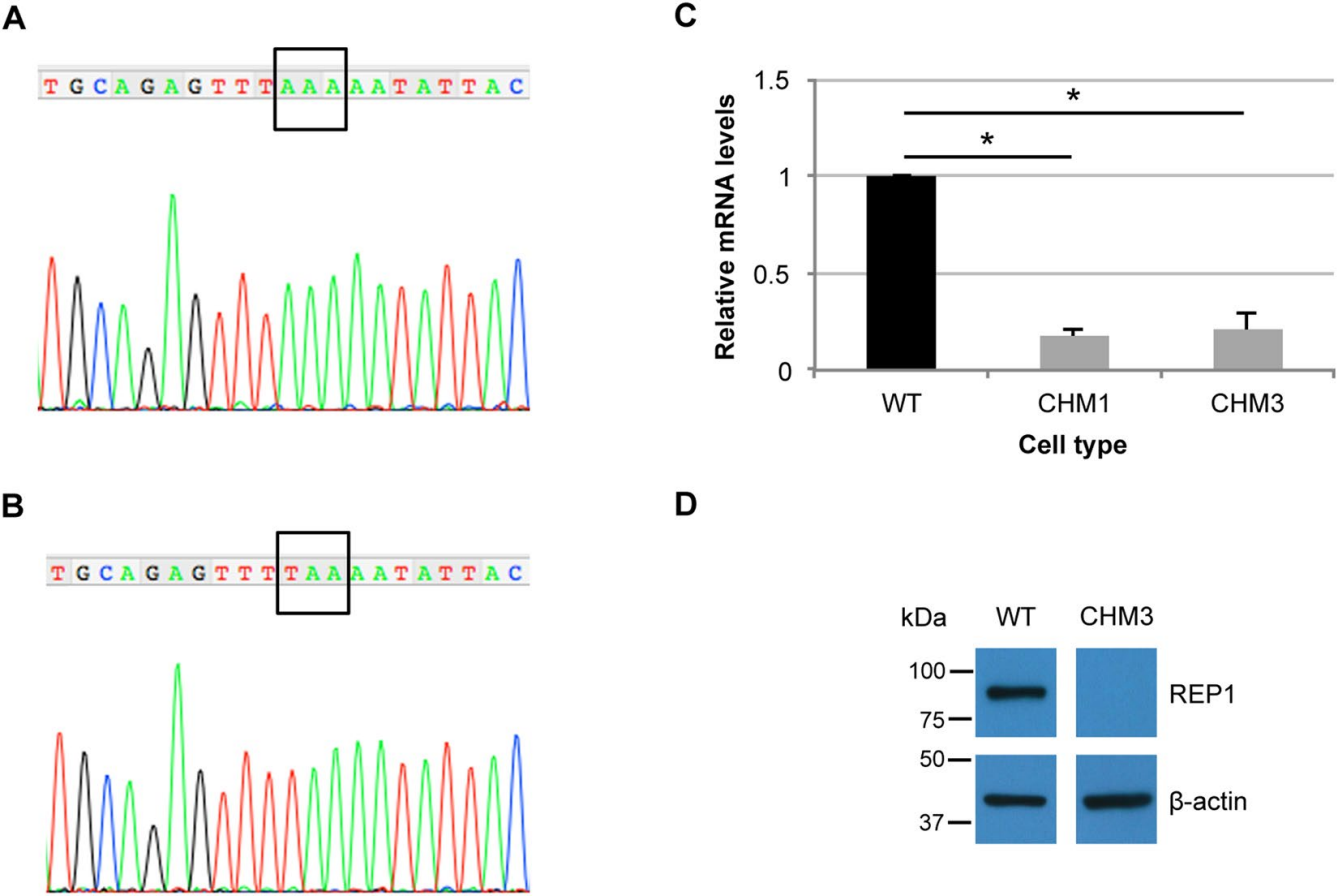
Effect of the c.772A>T variant in patient fibroblasts. (**A**) Sequence analysis of the genomic DNA of the wild-type *CHM* sequence from position c.762–782 shows the presence of an adenine at position c.772. (**B**) Sequence analysis of the same region in the genomic DNA of patient CHM3 shows the A>T transition at position c.772 resulting in a TAA codon. (**C**) qPCR analysis of *CHM* expression (in relative units) shows that the c.772A>T variant in CHM3 fibroblasts results in mRNA levels that are significantly reduced by 5-fold compared to wild-type (WT) cells and comparable to those of CHM1 fibroblasts. Data are expressed as mean ± SEM, *n* = 3; Mann and Whitney test *p < 0.05. (**D**) Western blot analysis shows the presence of REP1 in wild-type fibroblasts following hybridisation with a specific anti-REP1 antibody as compared to the absence of a detectable protein in CHM3 cells. Hybridisation with an antibody against β-actin serves as a loading control. Bands were cropped from the same gel with different exposure times for REP1 and β-actin.

We performed and cultured a skin biopsy of the patient CHM3, previously identified as carrying the variant c.772A > T in the *CHM* gene. This variant introduces a premature UAA stop codon at the position of a lysine residue, p.Lys258*, in REP1. We confirmed the presence of the c.772A > T variant in the emerging CHM3 fibroblasts by sequencing. In wild-type cells, c.772 corresponds to the first position of an AAA codon (Fig. 1A). In CHM3 cells, a T is found at the same position, which gives rise to a TAA codon in the DNA sequence (Fig. 1B). qPCR amplification using *CHM*-specific primers in exon 5 showed that the mRNA levels of CHM3 were significantly reduced to 20% of wild-type (*p* < 0.05; Fig. 1C), suggesting nonsense-mediated decay (NMD). The CHM3 mRNA levels were not significantly different to those of another patient, CHM1, who carries a deletion of exon 8, which results in a frame shift and a premature stop codon^28^. Lastly, the mutant p.Lys258* REP1 was not detected in CHM3 cells by western blot analysis (Fig. 1D), which was consistent with previous results for CHM1^28,29^.

Thus, the persistence of a *CHM* transcript in CHM3 was compatible with a translational read-through approach. Furthermore, the absence of REP1 expression represented a putative criterion to evaluate read-through.

### Effect of PTC124 treatment on CHM3 fibroblasts

**Figure 2:**
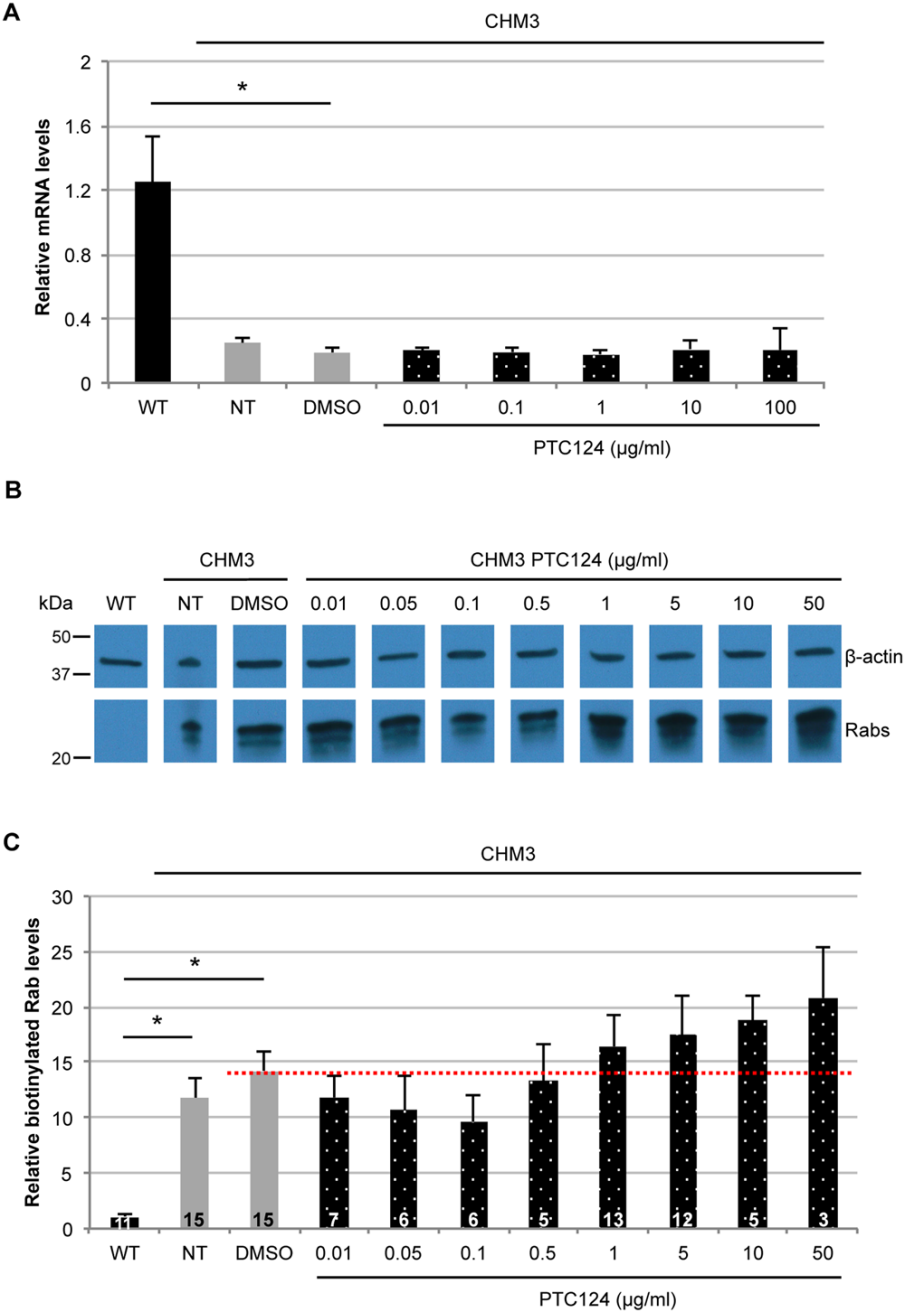
Effect of PTC124 treatment on CHM3 fibroblasts. (**A**) qPCR analysis of *CHM* mRNA levels in PTC124-treated patient fibroblasts. *CHM* transcript levels of CHM3 fibroblasts treated with DMSO (grey bar) remained significantly lower than wild-type levels (black bar). Data are expressed as mean ± SEM, *n* = 3; Mann and Whitney test *p < 0.05. Regardless of the dose of PTC124 used, *CHM* transcript levels (dotted bars) are not altered, as compared to DMSO-treated cells; Kruskal-Wallis test *p* > 0.05. NT = non-treated. (**B**) A representative *in vitro* prenylation assay showing biotinylated Rab proteins in wild-type (WT), and in non-treated (NT), DMSO-treated or PTC124-treated CHM3 fibroblasts. β-actin serves as a loading control. Bands were cropped from the same gel. (**C**) Quantification of the biotinylated Rab pool (in relative units), after normalisation to β-actin loading, in multiple independent assays shows a significant 10-fold increase in biotinylated Rabs in the non-treated (NT) and DMSO-treated fibroblasts of patient CHM3 (grey bars), as compared to wild-type (WT; black bar). Data are expressed as mean ± SEM; *n* is indicated in the corresponding histogram bars; Mann and Whitney test *p < 0.05. Treatment with a range of PTC124 doses did not significantly alter biotinylated Rab levels in CHM3 fibroblasts (dotted bars) as compared to DMSO-treated controls (red dotted line); Kruskal-Wallis test *p* > 0.05.

Initially, we tested for an effect of PTC124 treatment on *CHM* mRNA levels by qPCR analysis of fibroblasts. All working concentrations of PTC124 were kept at 0.2% DMSO, therefore, as a negative control, CHM3 cells were treated with 0.2% DMSO. Wild-type *CHM* mRNA levels remained significantly higher than DMSO-treated cells (*p* < 0.05; Fig. 2A). PTC124 treatment of CHM3 cells, regardless of the dose, did not significantly alter mRNA levels beyond that of DMSO alone (Fig. 2A). Consistently, REP1 was not detected by western blot analysis following treatment in the patient cell lysates (data not shown). To determine the limit of detection by western blot analysis, we performed serial dilutions of wild-type cell lysates and assayed for REP1. A progressively fainter REP1 band was observed down to a 1:10 dilution; a REP1 band could not be detected from 1:50 (Supplementary Fig. S1). This placed the limit of REP1 detection to between 2 and 10% of wild-type levels.

We tested if PTC124 treatment could improve the biochemical defect despite the absence of detectable REP1. We previously showed that the elevated levels of unprenylated cytosolic Rabs in CHM patients can be used as a read-out for therapy screening^28,29^. Unprenylated Rab levels can be detected using an *in vitro* prenylation assay, which relies on the addition of recombinant RGGTase, REP1, and biotinylated prenyl donor to the cytosolic cell fraction. If this fraction contains a Rab pool available for *in vitro* prenylation, the integrated biotin will be detected by western blot analysis using HRP-conjugated streptavidin. The quantity of biotinylated Rabs will thus be inversely proportional to endogenous REP1 activity^30^. We tested for a PTC124 effect on function by assaying for a reduction in the cytosolic Rab pool. One representative assay is shown in Fig. 2B, where wild-type fibroblasts show low levels of biotinylated Rabs in comparison to CHM3 fibroblasts. Quantification of multiple assays showed that the biotinylated Rab levels of non- or DMSO-treated CHM3 fibroblasts were significantly higher than wild-type (*p* < 0.05; Fig. 2C). A non-significant dose-dependent effect was observed following PTC124 treatment: Doses inferior to 0.1 µg/ml tended to decrease Rab levels and the most pronounced reduction was 30%; doses superior to 0.5 µg/ml tended to increase Rab levels.

Taken together, PTC124 treatment did not alter *CHM* mRNA levels or result in detectable REP1 expression. Depending on the dose, a non-significant decrease in cytosolic Rab levels was observed.

### Effect of NMD suppression on PTC124 treatment

**Figure 3:**
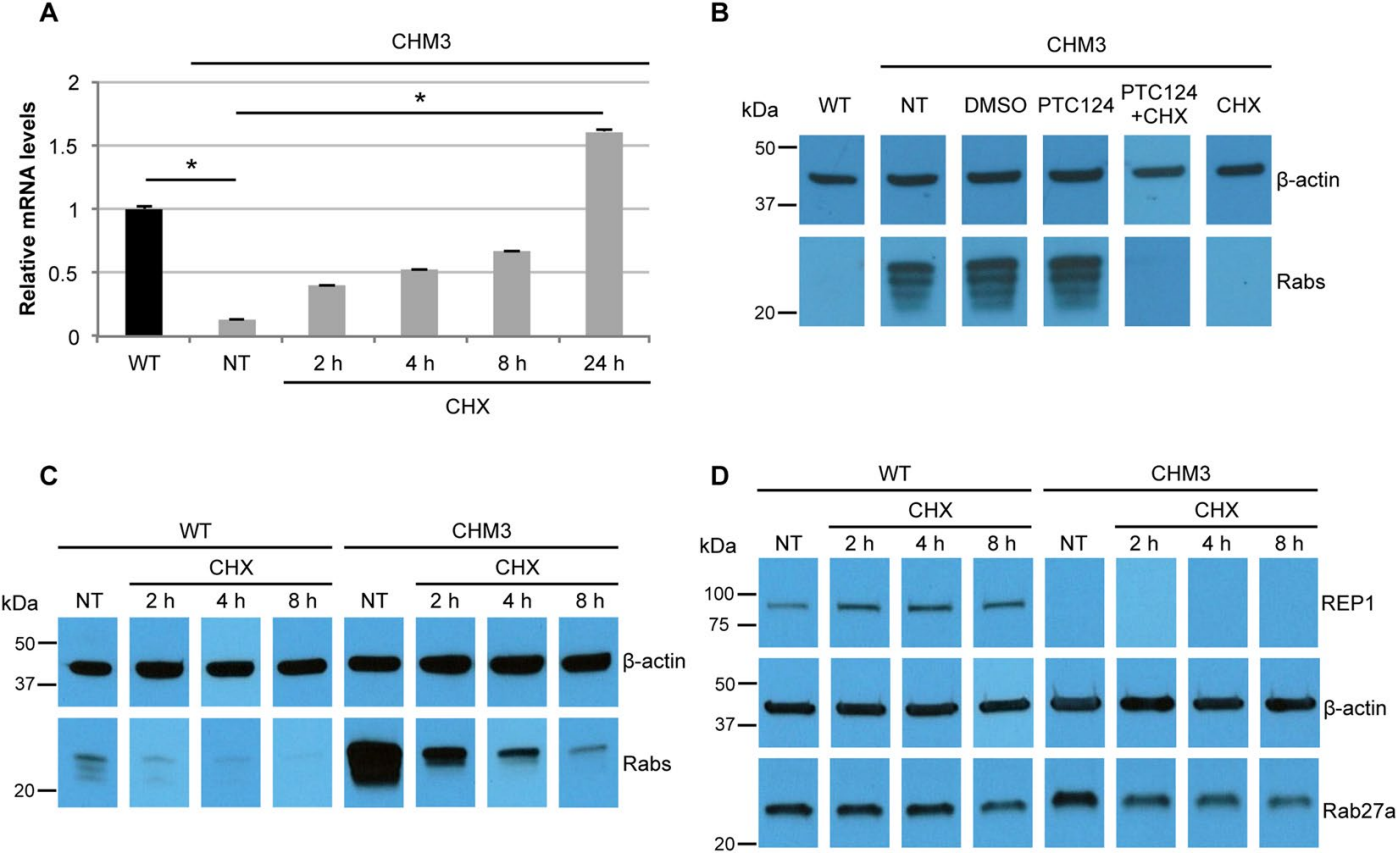
Effect of cycloheximide (CHX) treatment on fibroblasts. (**A**) qPCR analysis of *CHM* mRNA levels in CHX-treated CHM3 fibroblasts (grey bars). CHX treatment increases *CHM* transcript levels in a time-dependent manner, as compared to non-treated (NT) cells, with 24 h treatment resulting in significantly higher levels that exceed those of wild-type (WT; black bar). Data are expressed as mean ± SEM, *n* = 3; Kruskal-Wallis test *p < 0.05. (**B**) An *in vitro* prenylation assay using a biotinylated prenyl donor followed by western blot analysis shows a weak signal of incorporated biotin for the wild-type (WT) fibroblasts, as compared to the intense signal for the non-treated (NT), DMSO-treated or PTC124-treated CHM3 fibroblasts in the absence of CHX. By contrast, biotinylated Rab proteins cannot be detected in fibroblasts treated with CHX, coupled to or independent of PTC124 treatment. Bands were cropped from the same gel. (**C**) *In vitro* prenylation assay showing a time-dependent effect of CHX treatment on the biotinylated Rab population in both wild-type (WT) and CHM3 fibroblasts. Bands were cropped from the same gel. (**D**) Western blot analysis of CHX treatment on Rab27a expression in wild-type (WT) and CHM3 fibroblasts showing reduced expression levels from 8 h of CHX treatment in WT cells and from 2 h in CHM3 cells. REP1 expression in WT cells, as compared to CHM3 cells, is detected by hybridisation with an anti-REP1 antibody. In panels B–D, β-actin serves as a loading control. In panel D, bands were cropped from the same gel with a different exposure time for β-actin.

To determine whether PTC124 was more effective if mRNA levels were increased, we treated CHM3 fibroblasts with an NMD inhibitor, cycloheximide (CHX). qPCR analysis showed that CHX treatment increased *CHM* transcript levels, as compared to non-treated controls, proportionally to the incubation time (Fig. 3A). NMD was significantly inhibited (*p* < 0.05) after 24 h treatment. Combined CHX and PTC124 treatment of CHM3 cells abolished the biotinylated Rab population (Fig. 3B). However, the same effect was seen in cells treated with CHX alone. To exclude a side effect due to prolonged CHX treatment, we analysed shorter time courses on CHM3 and wild-type cells. A progressive reduction in biotinylated Rab levels in CHX-treated CHM3 cells was seen over time: 80% decrease at 2 h; 95% at 4 h, and 99% at 8 h (Fig. 3C). CHX-treated wild-type cells showed a similar time-dependent decrease: 60% at 2 h; 80% at 4 h, and 90% at 8 h. We also assayed the effect of lower CHX concentrations and a shorter time point in the presence and absence of PTC124, and observed a reduction in Rab levels for all conditions (Supplementary Fig. S2). Moreover, a decrease in Rab27a levels was detectable by western blot analysis, from 2 h in CHM3 cells and from 8 h in wild-type cells after CHX treatment (Fig. 3D). In contrast, REP1 and β-actin levels were unchanged.

In conclusion, NMD suppression by CHX could not be used to enhance read-through due to a nonsense mutation-independent effect of this compound on Rab expression.

### Generation of CHM3 iPSC-derived RPE

**Figure 4:**
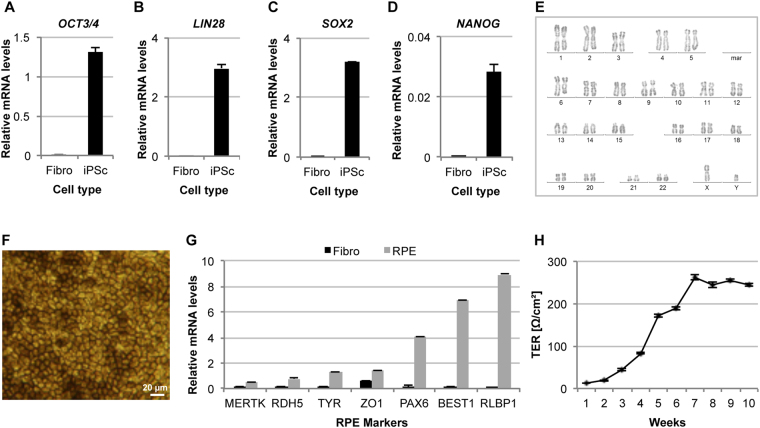
Generation of CHM3 iPSC and iPSC-derived RPE. qPCR analysis shows the mRNA levels of the host pluripotency markers *OCT3/4* (**A**), *LIN28* (**B**), *SOX2* (**C**) and *NANOG* (**D**) in CHM3 iPSC (black bars) as compared to the original fibroblasts. Data are expressed as mean ± SEM, *n* = 3. (**E**) Karyotype analysis of the CHM3 iPSC did not detect large chromosomal rearrangements. (**F**) The pigmented, cobblestoned appearance of the iPSC-derived RPE monolayer. (**G**) qPCR analysis of the expression of the typical RPE markers *MERTK, RDH5, TYR, ZO-1, PAX6, BEST1* and *RLBP1* shows an upregulation of expression (in relative units) in the CHM3 iPSC-derived RPE (grey bars) as compared to fibroblasts (black bars). Data are expressed as mean ± SEM, *n* = 3. (**H**) Weekly recordings of the transepithelial resistance (TER) following P3 seeding are presented as normalized Ω/cm^2^. Data are expressed as mean ± SEM, *n* = 3.

Although *CHM* is expressed ubiquitously, it is mainly the retina that shows a clinical phenotype. To determine whether a significant functional improvement with PTC124 could be obtained in a more clinically relevant cell type, we reprogrammed patient fibroblasts into iPSC for retinal differentiation. The iPSC were validated as pluripotent by qPCR analysis for the onset of expression of the host pluripotency genes *OCT3/4*, *LIN28*, *SOX2* and *NANOG* (Fig. 4A–D), and as genetically stable by karyotype analysis (Fig. 4E). iPSC were then differentiated into RPE, one of the primary tissues affected in CHM. We have previously shown that iPSC-derived RPE from CHM patients is morphologically and functionally characteristic of the RPE *in vivo*^28,29^. The CHM3 iPSC-derived pigmented monolayer (Fig. 4F) was assayed by qPCR analysis to verify the expression of RPE markers, which were upregulated as compared to fibroblasts (Fig. 4G). We also measured the transepithelial resistance (TER) to verify that the iPSC-derived RPE formed tight junctions. Consistent with ZO1 expression, the monolayer was confirmed as tight from 5 weeks post-differentiation when the TER values exceeded 150 Ω/cm^2^ (Fig. 4H). The TER values remained stable from 7 weeks post-differentiation.

Thus, we generated *bona fide* RPE for patient CHM3 to assay translational read-through in a more clinically pertinent cell type.

### Effect of PTC124 treatment on CHM3 iPSC-derived RPE

**Figure 5:**
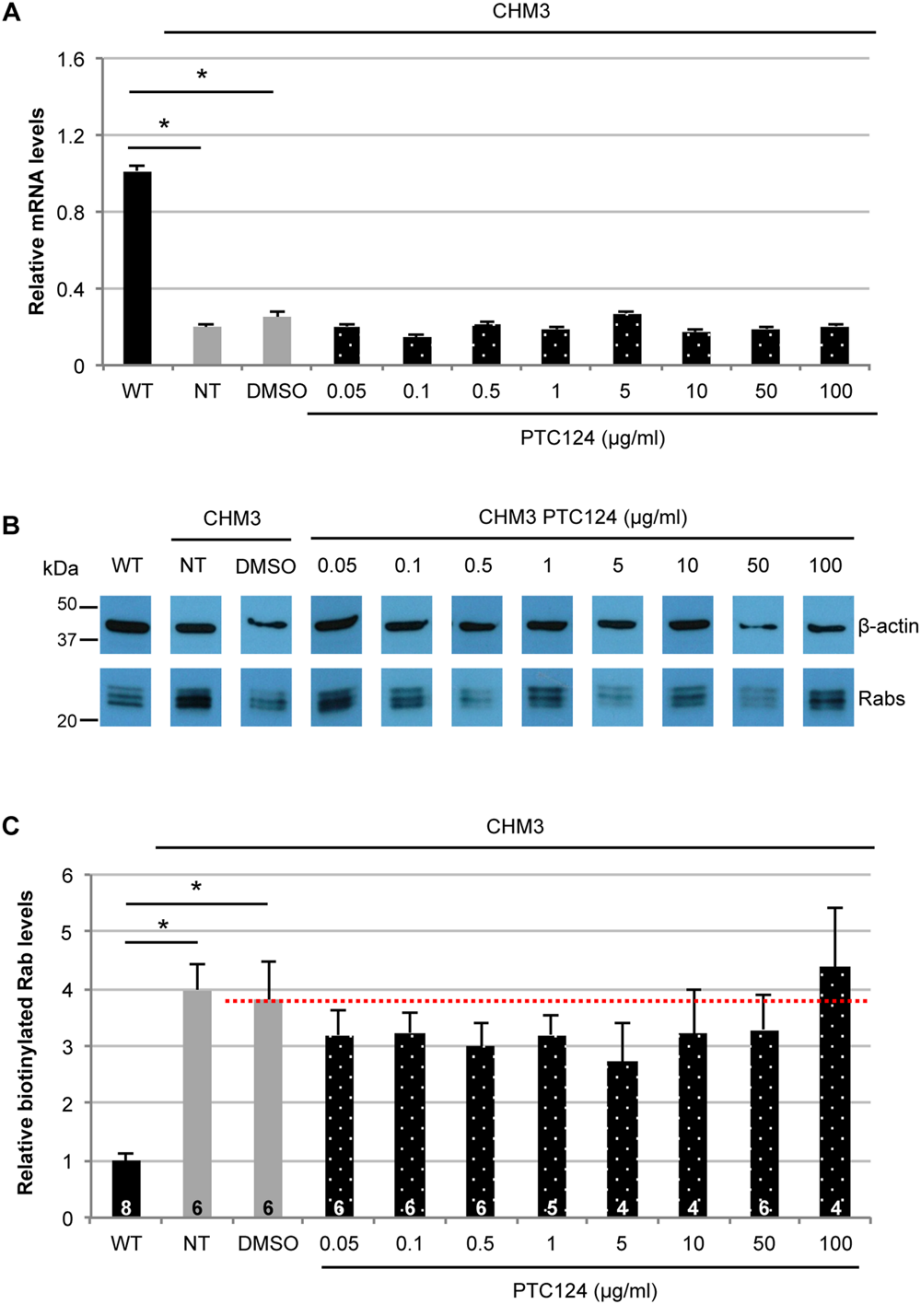
Effect of PTC124 treatment on CHM3 iPSC-derived RPE. (**A**) qPCR analysis of *CHM* mRNA levels in PTC124-treated patient iPSC-derived RPE (dotted bars). *CHM* transcript levels in non-treated (NT) and DMSO-treated CHM3 RPE (grey bars) are significantly lower than those of wild-type (WT; black bars) cells. Data are expressed as mean ± SEM, *n* = 3; Mann and Whitney test *p < 0.05. PTC124 treatment of CHM3 iPSC-derived RPE, regardless of the concentration used, did not significantly alter *CHM* transcript levels as compared to DMSO-treated cells; Kruskal-Wallis test *p* > 0.05. (**B**) A representative *in vitro* prenylation assay showing biotinylated Rab proteins in wild-type (WT), and in non-treated (NT), DMSO-treated or PTC124-treated CHM3 iPSC-derived RPE. β-actin serves as a loading control. Bands were cropped from the same gel. (**C**) Quantification of the relative biotinylated Rab levels in multiple independent assays, after normalisation to β-actin loading, shows a significant 4-fold increase in the non-treated (NT) or DMSO-treated iPSC-derived RPE of patient CHM3 (grey bars), as compared to wild-type RPE (WT; black bar). Data are expressed as mean ± SEM; *n* is indicated in the corresponding histogram bars; Mann and Whitney test *p < 0.05. Treatment with PTC124 results in a non-significant decrease in biotinylated Rab levels at all doses (dotted bars), with the exception of 100 µg/ml, as compared to DMSO-treated cells (red line); Kruskal-Wallis test *p* > 0.05.

We first assayed *CHM* mRNA levels in the iPSC-derived RPE by qPCR analysis before and after PTC124 treatment. In CHM3 RPE, mRNA levels were significantly lower than wild-type (equivalent to ~20%; *p* < 0.05; Fig. 5A), consistent with the observations on CHM3 fibroblasts (Fig. 1C). The mRNA levels of DMSO-treated cells remained significantly different from wild-type (*p* < 0.05). PTC124 treatment did not significantly increase expression levels as compared to DMSO-treated cells (Fig. 5A). We then tested for an effect of PTC124 treatment on function. One representative prenylation assay is shown in Fig. 5B, where it can be seen that wild-type iPSC-derived RPE had lower levels of biotinylated Rabs in comparison to untreated CHM3 RPE. Quantification of multiple assays showed that CHM3 iPSC-derived RPE had biotinylated Rab levels that were significantly higher than wild-type (*p* < 0.05; Fig. 5C). The biotinylated Rab levels of DMSO-treated CHM3 RPE remained significantly higher than wild-type (*p* < 0.05). Generally, all doses of PTC124, with the exception of the highest dose, showed a tendency to reduce biotinylated Rab levels, but this reduction was not statistically significant from DMSO-treated cells. The most pronounced reduction was a 30% decrease in Rab levels following treatment with 5 µg/ml PTC124.

To conclude, patient iPSC-derived RPE treated with PTC124 showed a non-significant decrease in cytosolic Rab levels at a larger range of doses than patient fibroblasts. However, the same 30% decrease was obtained in both cell types but at different doses.

### *In silico* prediction and 3D modelling of Lys258 substitutions

**Figure 6:**
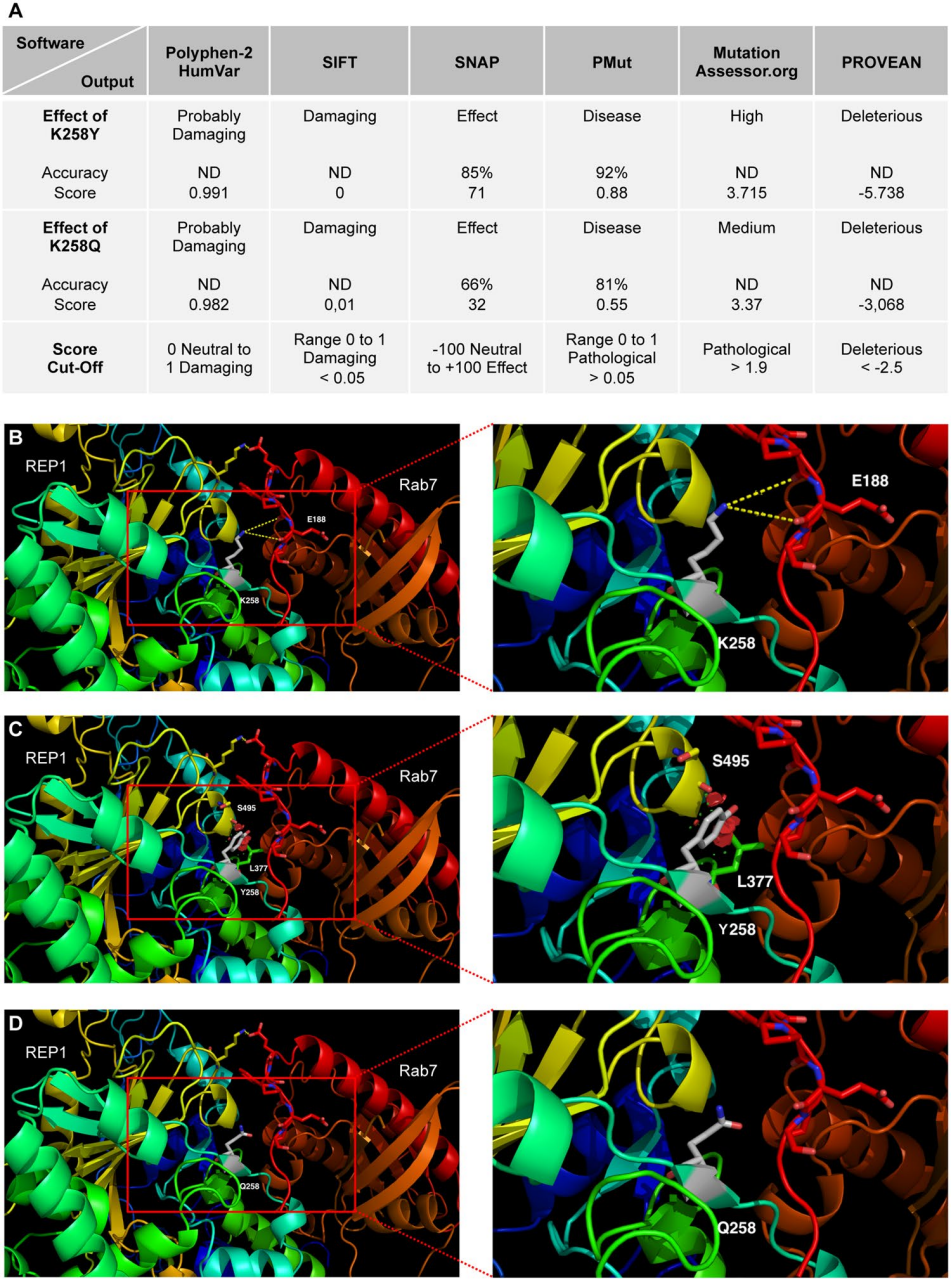
*In silico* analysis and 3D modelling of human REP1 and Rab7 interaction. (**A**) Predictions of various software on the impact of the amino acid substitutions p.Lys258Tyr (K258Y) and p.Lys258Gln (K258Q) on REP1 function. K258Y was predicted to be damaging by the software with relatively high accuracies and scores. K258Q was also predicted to be damaging at relatively lower accuracies and scores. ND = not determined. (**B**) The K258 residue (in grey) of REP1 may interact with the p.Glu188 (E188) residue of Rab7 via hydrogen bonds (yellow dots). (**C**) The introduction of a tyrosine at position 258 (Y258; in grey) is likely to induce a local rearrangement of REP1 due to steric hindrance (red areas around Y258) and would modify REP1-Rab7 binding. (**D**) The introduction of a glutamine at position 258 (Q258; in grey) is better tolerated, although an effect on REP1-Rab7 interaction could not be definitely ascertained.

The limited functional improvement in patient fibroblasts and iPSc-derived RPE suggested that PTC124 treatment, if mediating read-through, could not normalise the cell phenotype. It was shown that during translational read-through, not all amino acids are introduced at the target site with the same frequency^31^. In the case of PTC124-induced read-through of a UAA nonsense mutation in human cells, a tyrosine or a glutamine are introduced most frequently^32^. We therefore performed an *in silico* analysis to determine the effect of these substitutions on human REP1. Six different software predicted that the p.Lys258Tyr substitution is damaging to REP1 function (Fig. 6A). Moreover, we used a 3D model of REP1 and Rab7 interaction, and showed that Lys258 could interact directly with the Glu188 residue in the C-terminal part of Rab7 (Fig. 6B). This, and the observation that Lys258 of REP1 is highly conserved among evolutionarily diverse species (Supplementary Fig. S3), suggest a critical role. Consistently, on the 3D model, Tyr258 was predicted to induce a local rearrangement of the structure due to steric clashes (Fig. 6C) and to disrupt REP1-Rab7 interaction. *In silico* analysis predicted that the less frequent p.Lys258Gln substitution is also damaging to REP1 function, but with lower scores than the p.Lys258Tyr substitution (Fig. 6A). Analysis of the 3D model predicted that Gln258 was better tolerated, but we could not definitively ascertain if this substitution affected REP1-Rab7 interaction (Fig. 6D).

Therefore, if PTC124 read-through of p.Lys258* is taking place, the incorporation of a glutamine at this critical position may account for the limited improvement in the prenylation defect.

### Effect of proteasome inhibition concurrent to PTC124 treatment

**Figure 7:**
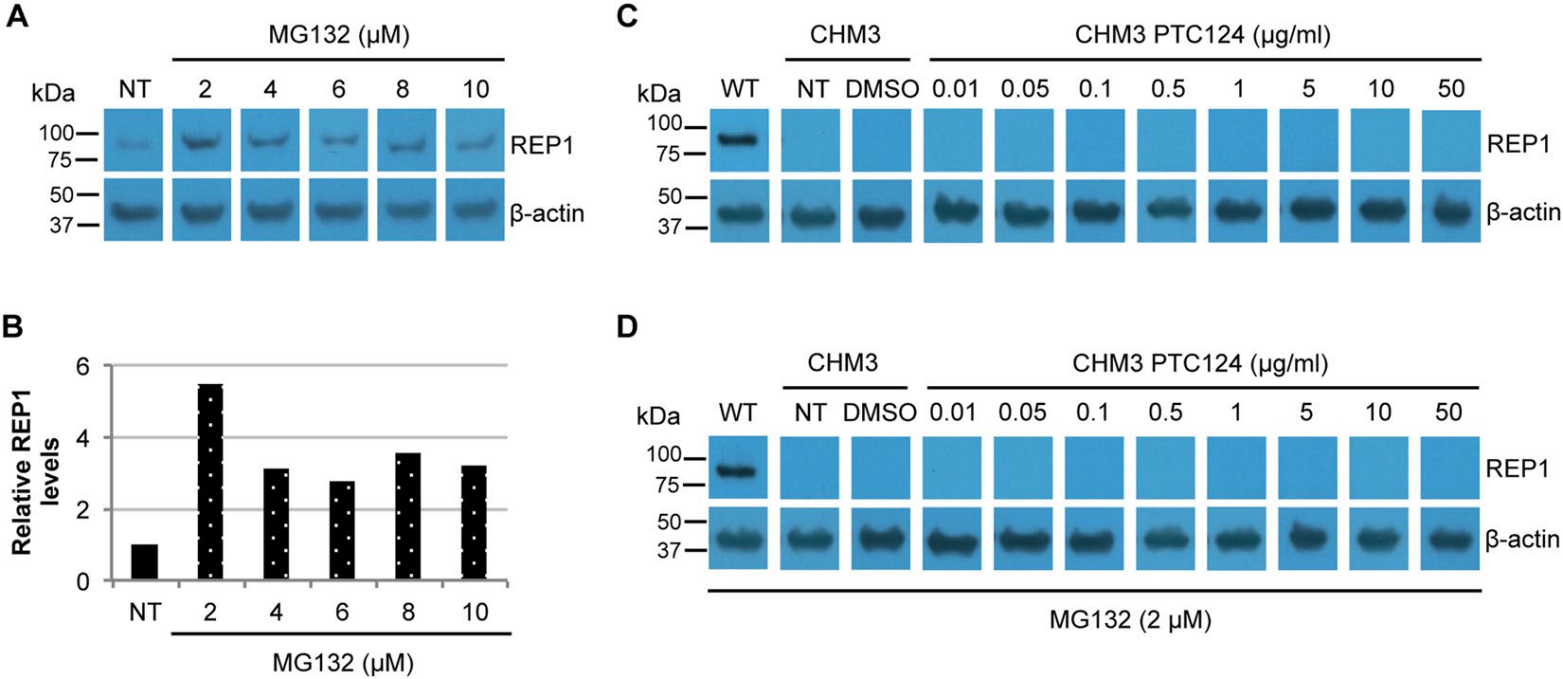
Effect of MG132 treatment on wild-type and CHM3 iPSC-derived RPE. (**A**) Western blot analysis of REP1 expression following treatment of wild-type iPSC-derived RPE with increasing concentrations of MG132. β-actin serves as a loading control. Bands were cropped from the same gel with different exposure times for REP1 and β-actin. (**B**) Quantification of REP1 expression in panel A shows an increase at all concentrations used with the highest levels observed at 2 µM (~5-fold higher than non-treated (NT) cells). Western blot analysis of REP1 expression in non-treated (NT), DMSO-treated or PTC124-treated CHM3 iPSC-derived RPE in the absence (**C**) or presence (**D**) of MG132. β-actin serves as a loading control. No expression could be seen in CHM3 iPSC-derived RPE as compared to wild-type (WT) RPE regardless of the treatment used. In panels C and D, bands were cropped from the same gel with different exposure times for REP1 and β-actin.

As we observed a non-significant trend to functional improvement in iPSC-derived RPE, we tested for REP1 expression in PTC124-treated cells by western blot analysis. In parallel, we tried to enhance detection levels by treating the RPE with a commonly used proteasome inhibitor, MG132, to prevent degradation of a mutant REP1 potentially produced post-read-through. The working MG132 concentration was determined by assaying for an increase in wild-type REP1 expression by western blot analysis. Following 24 h-treatment, all MG132 concentrations tested increased REP1 levels compared to untreated cells (Figs. 7A), and 2 µM resulted in the highest levels (Fig. 7B); proteasome inhibition was confirmed by assaying for an increase in poly-ubiquitinylated conjugates (data not shown). REP1 expression was then assayed by western blot analysis of CHM3 iPSC-derived RPE treated with PTC124 both in the absence (Fig. 7C) and presence (Fig. 7D) of MG132. Firstly, REP1 expression was not detected in non- or DMSO-treated CHM3 cells as compared to wild-type cells. Secondly, PTC124 treatment did not result in the detection of REP1 in CHM3 RPE (Fig. 7C). Lastly, MG132 used in conjunction with PTC124 did not improve the detection levels of REP1 in treated CHM3 cells (Fig. 7D).

Taken together, although a non-significant functional improvement was detected with PTC124 in iPSC-derived RPE, we could not detect the expression of REP1 even following inhibition of the proteasome.

### Effect of PTC124 treatment using a UAA-mutated *CHM* overexpression system

**Figure 8:**
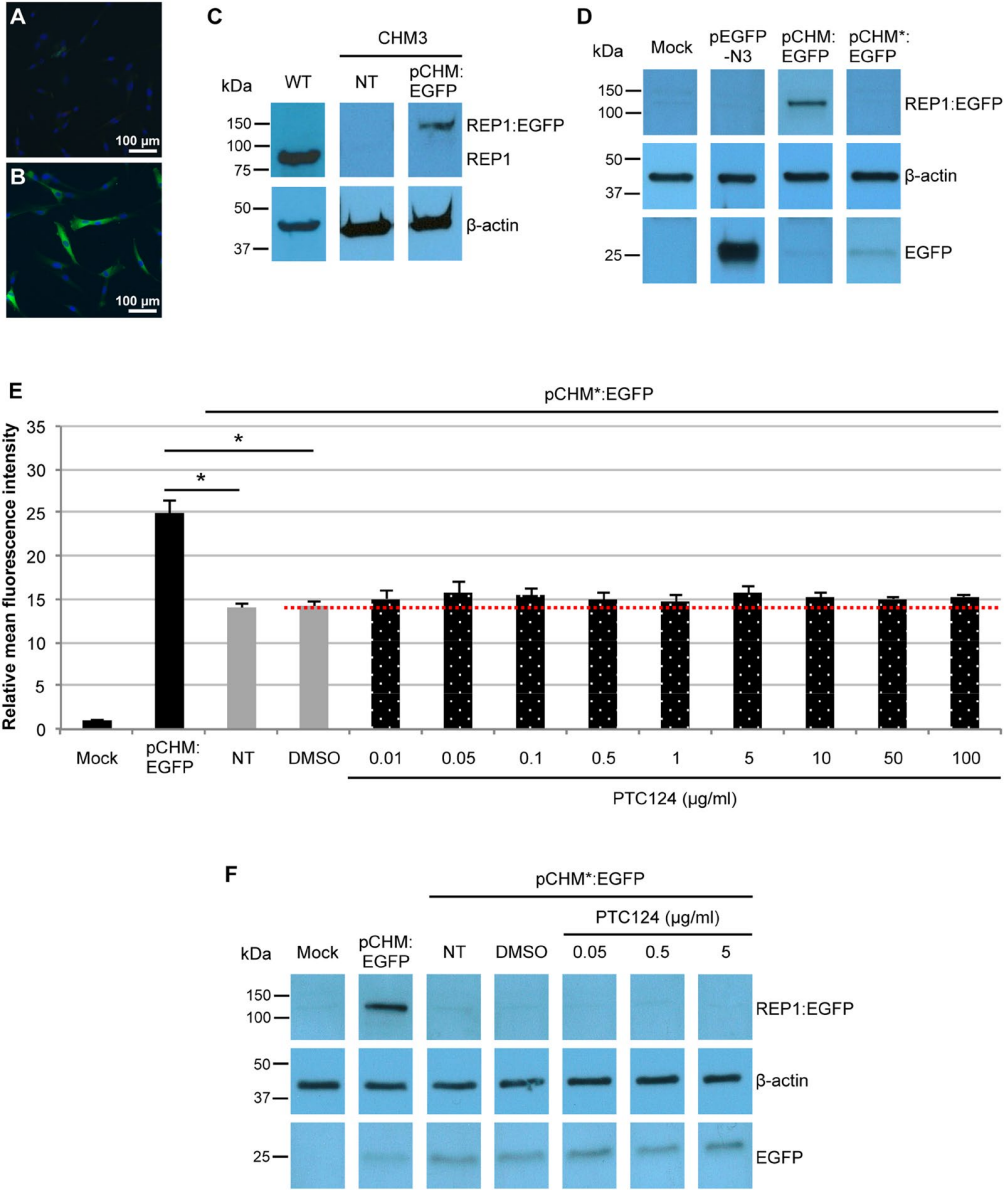
Effect of PTC124 treatment on a UAA-mutated *CHM* overexpression system. Immunofluorescence studies with an anti-GFP antibody of mock-transfected (**A**) and pCHM:EGFP-transfected (**B**) CHM3 fibroblasts showing EGFP expression (in green; nuclei in blue). (**C**) Western blot analysis of transfected CHM3 fibroblasts using an anti-REP1 antibody. An ~90 kDa REP1 band is detected in wild-type (WT) cells but not in mock-transfected cells. A larger (~120 kDa) REP1:EGFP band is seen in pCHM:EGFP-transfected cells. β-actin serves as a loading control. The WT and CHM3 lanes were cropped from two different gels. (**D**) Western blot analysis of transfected COS-7 cells using an anti-EGFP antibody. No signal can be detected in mock-transfected cells. An ~25 kDa EGFP band can be detected in cells transfected with the empty pEGFP-N3 plasmid. An ~120 kDa REP1:EGFP band is seen in pCHM:EGFP-transfected cells but not in pCHM*:EGFP-transfected cells; a faint ~25 kDa EGFP band is detected in both. Bands were cropped from the same gel with different exposure times for EGFP and β-actin. (**E**) Flow cytometry analysis of the mean fluorescence intensity of transfected COS-7 cells treated with various doses of PTC124. A 25- and 14-fold increase in fluorescence is seen in cells transfected with pCHM:EGFP (black bar) and pCHM*:EGFP (grey bars), respectively, as compared to mock-transfected cells. There was a significant 1.8-fold difference in intensity in non- and DMSO-treated pCHM*:EGFP-transfected cells as compared to pCHM:EGFP-transfected cells. Data are expressed as mean ± SEM, *n* = 2; Mann and Whitney test *p < 0.05. These levels did not significantly increase following PTC124 treatment (dotted bars) as compared to DMSO-treated controls (red dotted line); Kruskal-Wallis test *p* > 0.05. NT = non-treated. (**F**) Western blot analysis of transfected COS-7 cells following treatment with PTC124 using an anti-EGFP antibody. No signal is detected in mock-transfected cells. An ~120 kDa REP1:EGFP band is seen in pCHM:EGFP-transfected cells but not in non-treated (NT), DMSO-treated or PTC-124-treated pCHM*:EGFP-transfected cells. An ~25 kDa EGFP band is detected in all transfected cells, which is more intense for the pCHM*GFP-transfected cells. β-actin serves as a loading control. Bands were cropped from the same gel with different exposure times for β-actin.

Due to the undetectable expression of REP1 in PTC124-treated patient fibroblasts and iPSC-derived RPE, we generated an overexpression system to obtain higher REP1 levels. To this end, the *CHM* ORF, without the native 3′ stop codon, was cloned upstream of the ATG initiation codon of *EGFP* in the expression plasmid pEGFP-N3 to generate the fusion plasmid pCHM:EGFP. As compared to non-transfected cells (Fig. 8A), EGFP expression can be seen in CHM3 fibroblasts transfected with pCHM:EGFP by immunofluorescence studies (Fig. 8B). Western blot analysis showed that REP1 was not detected in non-transfected  CHM3 fibroblasts whereas REP1:EGFP was detected in transfected cells (Fig. 8C); REP1:EGFP was larger than native REP1 detected in wild-type fibroblasts. By site-directed mutagenesis, we then introduced the c.772A>T mutation into pCHM:EGFP, generating the UAA nonsense plasmid pCHM*:EGFP. Western blot analysis of transfected COS-7 cells detected REP1:EGFP in pCHM:EGFP-transfected cells but not in pCHM*:GFP-transfected cells (Fig. 8D). Furthermore, a fainter band corresponding to EGFP alone could be detected in all transfected cells, as compared to cells carrying the empty pEGFP-N3.

Transfected COS-7 cells were then treated with various PTC124 doses and assayed for mean EGFP fluorescence intensity by flow cytometry (Fig. 8E). Firstly, pCHM:EGFP-transfected cells showed high EGFP levels compared to mock-transfected cells. Secondly, EGFP levels were moderately high in non- or DMSO-treated cells transfected with pCHM*:EGFP; consistent with the expression of EGFP alone detected by western blot analysis (Fig. 8D). Nonetheless, these EGFP levels were significantly lower than those of pCHM:EGFP-transfected cells (p < 0.05). Thirdly, PTC124 treatment of pCHM*:GFP-transfected cells did not significantly increase EGFP levels as compared to DMSO-treated controls, regardless of the dose. Lastly, western blot analysis of transfected COS-7 cells treated with three representative PTC124 doses did not result in detectable REP1:EGFP production (Fig. 8E). This confirmed that the background EGFP levels were not masking the detection of a potential REP1:EGFP fusion protein produced post-read-through.

In conclusion, detection of a REP1 protein following PTC124 treatment of the CHM3 nonsense mutation was not improved using an artificial overexpression assay.

## Discussion

IRDs lend themselves to different therapeutic possibilities, such as TRID therapy in the case of nonsense mutations^33^. In this study, we assayed the potential for one particular TRID, PTC124, to correct the functional defect of the IRD CHM. CHM is an interesting target because a high percentage of causative *CHM* mutations are nonsense mutations^20^. PTC124 was recently shown to have a positive effect on the phenotype of the CHM zebrafish model, carrying a UAA nonsense mutation that gives rise to a p.Gln33* alteration^25^. We thus performed a complementary study on patient cells carrying a UAA mutation. This mutation is situated in exon 6 of *CHM* and gives rise to the p.Lys258* alteration.

PTC124 treatment of patient fibroblasts did not significantly alter *CHM* mRNA levels or biotinylated Rab levels. A tendency for a dose-dependent response was however noted; the best response was seen with 0.1 µg/ml PTC124, which mediated a 30% reduction. Interestingly, a significant 40% reduction in biotinylated Rab levels was observed in a previous study on CHM fibroblasts carrying the UAG nonsense mutation p.Tyr42* following treatment with ~10 µg/ml PTC124^27^. Here, concentrations >1 µg/ml tended to increase Rab levels. It was previously proposed that the type of nonsense mutation (UGA>UAG>UAA) and immediately downstream nucleotide (C>U>A≥G) could significantly impact TRID efficiency^34^. The p.Lys258* mutation is a UAA nonsense mutation followed by an A nucleotide. This could explain why the p.Tyr42* cells responded better to PTC124 than the p.Lys258* cells, as they carry a UAG nonsense mutation followed immediately by a U nucleotide. However, the CHM zebrafish model also carries a UAA mutation followed by an A nucleotide, like p.Lys258*, and it showed an even better response to PTC124 treatment than p.Tyr42* cells, with a normalisation of the prenylation defect. Therefore, our data suggest that the type of premature stop codon or surrounding sequence might not be the only factors accounting for the differences in PTC124 efficacy, at least in the case of CHM.

We tried to improve PTC124 efficiency by blocking NMD. Regardless of the concentration or incubation time, CHX treatment caused a disappearance of the Rab pool independently of PTC124 in both wild-type and patient fibroblasts. The effect was thus independent of a nonsense mutation and NMD inhibition. In addition, a CHX-induced decrease in Rab27a expression was also observed, suggesting that CHX treatment had an effect on protein synthesis, as previously reported^35^. This decrease was more pronounced in patient cells as compared to wild-type, maybe because, in the latter, the majority of Rab27a is prenylated and membrane-associated, and hence more stable. By contrast, in patient cells, a large proportion of Rab27a is unprenylated and thus more susceptible to degradation. This is consistent with the observation that CHX treatment did not affect the expression of REP1, which is present as part of the prenylation complex in wild-type cells, or β-actin, which is part of the cytoskeleton complex in wild-type and patient cells. In a similar manner, the dual activity of CHX negatively impacted the functional studies of long QT syndrome 2 due to nonsense mutations in the gene *hERG*^36^.

As the retina is the main tissue type to show clinical signs in patients, it has been hypothesised that REP1 activity is more critical in this tissue^37^. Therefore, PTC-induced functional improvement may be dependent on the cell type, as was shown for other diseases^38^. We thus generated a pertinent human cell model by reprogramming the UAA-carrying fibroblasts into iPSC, which were differentiated into RPE, one of the primary tissues affected in CHM. PTC124 treatment of the iPSC-derived RPE resulted in a non-significant decrease of biotinylated Rabs at a large range of doses; the best response was seen with 5 µg/ml, which decreased Rabs by 30%. Therefore, if PTC124 treatment was performing read-through of the UAA nonsense mutation in fibroblasts and iPSC-derived RPE, it could not normalise the cellular phenotype.

This may not be surprising, as it has been shown that not all amino acids are introduced into the target site at the same frequency during translational read-through^31^. Therefore, the wild-type residue will only be inserted a percentage of the time, if at all. In the case of PTC124-induced translational read-through of a UAA nonsense mutation, a tyrosine (~60% of cases) or a glutamine (~40%) is introduced^32^. However, both substitutions were predicted to be relatively damaging to human REP1 function by *in silico* analysis. Moreover, 3D modelling predicted that Lys258 of REP1 interacts with Rab7. This critical role is consistent with its high conservation among evolutionarily diverse species. Consistently, Tyr258 was predicted to disrupt REP1-Rab7 interaction but the effect of Gln258 could not be ascertained. Taken together, in the majority of cases, PTC124 read-through of p.Lys258* would produce a deleterious mutant protein.

It is tempting to speculate that Gln258 may allow residual REP1 function that could account for the decrease in Rab levels, but that was not sufficient to reach significance. Along this line, in the case of a UAG nonsense mutation, a glutamine (~53%), tyrosine (~44%) or tryptophan (~1%) residue are incorporated most frequently^32^. Therefore, for the p.Tyr42* mutation, the wild-type amino acid has a relatively high chance of being incorporated following read-through, which could account for the significant prenylation rescue observed post-PTC124 treatment^27^. Similarly, in the case of the p.Gln33* UAA nonsense mutation carried by the CHM zebrafish model, the wild-type glutamine would be incorporated relatively frequently. Moreover, the Gln33 residue is not evolutionarily conserved suggesting that non-wild-type substitutions, such as a tyrosine, could also be relatively well-tolerated^26^. This cumulative effect could explain the normalisation of the biochemical phenotype following PT124 treatment of the *chm*^*−/y*^ zebrafish larvae^27^.

In general, REP1 tolerance to amino acid changes is still in debate. If we take into account genotype datasets of large populations such as ExAC^39^, the expected (116) and observed (112) number of *CHM* missense variants is highly similar (see http://exac.broadinstitute.org/gene/ENSG00000188419). The majority of these variants are considered to be clinically neutral. Interestingly, the first pathogenic missense *CHM* variant was reported in 2009^40^ whereas gene screening began 20 years prior^41^. To date, nine CHM-associated variants have been reported (see http://www.lovd.nl/CHM), and only three have been proven to be pathogenic^29,40,42^. This low number remains unexplained. Particularly pertinent for this study, is that the missense-encoded mutant REP1 is not always detected by western blot analyses suggesting this protein is highly sensitive to certain modifications^40^. This could be paralleled to the lack of a detectable REP1 following PTC124 treatment of p.Lys258*-carrying and p.Tyr42*-carrying cells. We show here that the detection limit of western blot analysis for REP1 is between 2 and 10% of wild-type levels. Therefore, this implies that, despite the significant functional rescue in the p.Tyr42* study, REP1 levels were below 10%. It is questionable that such low levels could give rise to a clinical benefit if we consider that, in the zebrafish model, the residual maternal rep1 derived from the persistent yolk sac in 6 dpf larvae is estimated at ~15% and this is not sufficient to mediate normal development and prevent lethality^27^.

To increase the REP1 detection threshold following PTC124 treatment of p.Lys258* iPSC-derived RPE, we inhibited the proteasome prior to western blot analysis, but still did not detect the protein. We therefore designed an artificial overexpression system containing the same mutation carried by the patient cells to increase REP1 levels. By fusing *EGFP* to the mutant *CHM* gene, we used flow cytometry and western blot analysis to assay for REP1:EGFP production post-treatment. The fusion construct showed high background fluorescence levels, which were due to the expression of EGFP alone, however PTC124-treatment did not significantly increase fluorescence beyond background. Furthermore, a REP1:EGFP protein could not be detected by western blot analysis. Therefore, in the absence of detectable REP1, it is difficult to conclude that the effect observed post-PTC124 treatment of p.Lys258* is due to read-through of the nonsense mutation.

To date, the efficiency of PTC124 has been highly controversial^43^. In the case of Duchenne muscular dystrophy^15^, cystic fibrosis^44^, Usher syndrome 1C^45^, retinitis pigmentosa^17,18^ and anirida^19^, PTC124 was suggested to improve cellular phenotype. By contrast, in the case of long-QT syndrome type 1^46^, lysosomal storage disorders^47^, conduction disease and potential lethal arrhythmias^48^, infantile neuronal ceroid lipofuscinosis^38^ and leptin receptor-associated obesity^49^, PTC124 was ineffective. It may be that the answer to these discrepancies could lie in the characteristics of the target amino acid. This may also explain why only a subgroup of patients in the Duchenne muscular dystrophy phase 3 clinical responded to PTC124 treatment^50^.

In conclusion, in the case of the p.Lys258* mutation, we do not have firm evidence that PTC124 did accomplish read-through. However, if read-through were taking place, we highlight other considerations such as conservation or type of the target amino acid that may prevent the production of a functional protein. This may suggest that different nonsense mutations for the same gene might be less prone to read-through, in agreement with other studies^51^. Therefore, to render TRID therapy more effective, a personalised, rather than a widespread, approach may need to be considered, whereby patient cells are screened *in vitro* for drug efficiency prior to inclusion in a clinical trial.

## Methods

### Skin biopsy and fibroblast culture

The skin biopsy of a 10 year-old boy (CHM3) carrying a UAA nonsense mutation was performed at the Centre of Reference for Genetic Sensory Disorders (CHRU Montpellier, France) according to local guidelines and regulations. Informed consent was obtained from the subject’s mother. All experimental protocols were approved by the National Agency for Health and Medicine under the biomedical research authorisation number 2014-A00549-38. The corresponding fibroblasts were cultured in AmnioMAX C100 basal media with GlutaMAX (Gibco, ThermoFisher Scientific, Villebon sur Yvette, France) containing 10% decomplemented foetal calf serum (FCS; Lonza, Verviers, Belgium), 1% penicillin-streptomycin-amphotericin B (Lonza) and 2% AmnioMax-C100 supplement (Gibco) at 37 °C under 5% CO_2_^30^.

### Generation of iPSC

The CHM3 fibroblasts were seeded on day (D) −1 at a density of 2 × 10^5^ cells per 9.4 cm^2^ in high glucose DMEM containing GlutaMAX and sodium pyruvate (Gibco) and supplemented with 10% FCS, 1% non-essential amino acids (Gibco), 1 mM L-ascorbic acid (Sigma-Aldrich, Saint Quentin Fallavier, France) and 10 ng/ml bFGF (Peprotech, Neuilly Sur Seine, France). On D0, cells were reprogrammed using the CytoTune-iPS 1.0 Reprogramming kit (Life Technologies, ThermoFisher Scientific) containing four Sendai virus-based reprogramming vectors expressing *OCT4*, *SOX2*, *KLF4* and *c-MYC* at an MOI of 3. On D1, the media was refreshed, and, on D5, the transduced fibroblasts were passaged onto feeder cells (prepared as described^28^) at a density of 10^5^ cells per 9.4 cm^2^. On day 6, the media was changed to ES media: Knockout DMEM (Gibco) supplemented with 20% KO serum replacement (Gibco), 200 mM GlutaMAX (Gibco), 1% non-essential amino acids, 0.1% β-mercaptoethanol (Gibco), 1% penicillin-streptomycin (Gibco) and 10 ng/ml bFGF, and (until D11) 500 µM Valproic acid (Sigma-Aldrich). Resulting iPSC were mechanically passaged and subsequently adapted to feeder-free conditions on a 1/100 dilution Corning Matrigel HESC-qualified matrix (Analytic Lab, St Mathieu de Treviers, France) and in Essential (E) 8 media (Gibco). Passages were subsequently performed using Versene solution (Gibco).

### Karyotype analysis

iPSC were grown to 50% confluence, treated with 0.1% Nocodazole (Sigma-Aldrich) and the pellet prepared as previously described^29^. Karyotype analyses were performed by the Chromostem facility (CHRU Montpellier, France).

### Generation of iPSC-derived RPE

The iPSC were grown to confluence and the E8 media was changed to bFGF-depleted ES media to initiate spontaneous differentiation. Pigmented foci were manually dissected, dissociated and seeded as previously described^29^. For TER measurements, iPSC-derived RPE was seeded on Matrigel-coated BD Falcon cell culture inserts (Dominique Dutscher, Brumath, France). TER was measured using the Epithelial Volt/Ohm Meter EVOM2 (World Precision Instruments, Hertfordshire, U.K.) as described^29^.

### Mutation verification

To verify the presence of the c.772A>T variant situated in exon 6 in cultured cells, genomic DNA was isolated using the DNeasy Blood & Tissue Kit (Qiagen, Les Ulis, France) according to the manufacturer’s instructions. A 768-bp product was then amplified using the F primer 5′-GGG TAG GGA GGT TCA CTG GA-3′ and the R primer 5′- AAA TCA CCA CGG AGG ACT GG-3′. This amplicon was cleaned using the ExoSAP-IT PCR Clean-up Kit (GE Healthcare, Velizy Villacoublay, France) prior to sequencing with the F primer 5′-ATC TTT ATT GTT TTC CAA TT-3′ and the BigDye Terminator Cycle Sequencing Ready Reaction kit V3.1 on a 3130xL Genetic Analyser (Applied Biosystems, Foster City, CA).

### Reverse transcription and quantitative PCR studies

RNA was isolated and 150 ng was reverse transcribed as described^29^. Quantitative RT-PCR (qPCR) was used to analyse the expression of endogenous *CHM* in fibroblasts and iPSC-derived RPE, and the endogenous pluripotency genes *OCT3/4*, *LIN28*, *SOX2* and *NANOG* in iPSC, using the previously described primers^28^. qPCR was used to analyse the expression of the RPE genes *MERTK*, *RDH5*, *TYR*, *ZO1*, *PAX6*, *BEST1* and *RLBP1* in iPSC-derived RPE, and primer sequences are available upon request. All results were normalised to *GAPDH* expression. Reactions were performed using the LightCycler® 480 SYBR Green I Master mix on a LightCycler® 480 II thermal cycler (Roche). Results were analysed using LightCycler® 480 software and the Microsoft Excel programme.

### Generation of pCHM:EGFP fusion constructs

The wild-type *CHM* ORF was amplified using the *Sac*I site-containing F primer 5’- GAG CTC ATG GCG GAT ACT CTC CCT TC-3’ and the R primer 5′-GGT ACC TTC AGA GGA CTC CTC TAG GT-3′, containing a *Kpn*I site at the place of the *CHM* stop codon, and subcloned using the pGEM-T Easy vector system (Promega, Charbonnières-les-Bains, France). Following digestion with the restriction enzymes *Sac*I and *Kpn*I, the *CHM* ORF was then subcloned into the Clontech pEGFP-N3 expression plasmid (Ozyme, Saint-Quentin-en-Yvelines, France) to generate the pCHM:EGFP fusion construct. The c.772A>T variant was then introduced into the wild-type plasmid to generate the plasmid pCHM*:EGFP using the Stratagene QuikChange site-directed mutagenesis kit (Agilent, Les Ulis, France) and the F primer 5′-GTT AGT CGA TAT GCA GAG TTT TAA AAT ATT ACC AGG ATT CTT G-3′, with its reverse complementary primer.

### Immunofluorescence studies

One million CHM3 fibroblasts were nucleofected with 1 µg DNA of the pCHM:EGFP fusion plasmid and 100 µl of P2 Primary Cell 4D-Nucleofector solution (Lonza, Levallois-Perret, France) using the program EN-150 of the Amaxa 4D-Nucleofector according to the manufacturer’s instructions. Non-transfected and transfected cells were then seeded in four 1.9 cm^2^ wells containing glass coverslips. Media was refreshed the following day and, 72 h post-nucleofection, cells were fixed with 4% Alfa Aesar paraformaldehyde (PFA; ThermoFisher Scientific), blocked in 5% FCS and 1% bovine serum albumin (BSA; Sigma-Aldrich), and permeabilised with 0.2% Triton X-100 (Sigma-Aldrich). The coverslips were then incubated with a 1:200 dilution of rabbit anti-GFP antibody (Invitrogen-Molecular probes) overnight at 4 °C and with a 1:1000 dilution donkey anti-rabbit IgG-Alexa Fluor 488 secondary antibody containing 0.2 µg/ml bisBenzimide Hoechst (Sigma-Aldrich) for 45 min at room temperature prior to mounting in Dako Fluorescent Mounting Media (Dako France SAS, Les Ulis, France).

### Drug treatments

PTC124 was purchased from Selleck Chem (Euromedex, Souffelweyersheim, France) and dissolved in DMSO (ThermoFisher Scientific) to a stock concentration of 50 mg/ml. Cells were treated in media without antibiotics at various working concentrations (ranging from 0.01 µg/ml to 100 µg/ml) but always at the same final concentration of DMSO (0.2%) as the mock-treated controls (treated with DMSO alone). Cells were treated for 24 h and dosed twice in that period. In the case of fibroblasts, cells were generally seeded at 2 × 10^5^ cells per 9.4 cm^2^ and treated 24 h post-seeding. iPSC-derived RPE was used at passage 3 upon maturity (from ~8 weeks post-differentiation). To increase *CHM* mRNA levels, fibroblasts were treated with 20, 100 or 500 µg/ml cyclohexamide (Sigma-Aldrich) for 2 to 24 h in the absence or presence of PTC124. To increase REP1 levels, iPSC-derived RPE was treated with 2 to 10 µM MG132 (Tocris Bioscience, Lille, France) in the absence or presence of PTC124 for 24 h.

### Transfection and flow cytometry

COS-7 cells were seeded at a density of 7.5 × 10^4^ cells per 1.9 cm^2^ in DMEM containing GlutaMAX (Gibco) supplemented with 10% FCS. Twenty-four h post-seeding cells were transfected using 1 µg DNA of the pCHM:EGFP fusion plasmids and 1.25 µl Lipofectamine 3000 transfection reagent (Thermo Fisher Scientific, Courtaboeuf, France) according to the manufacturer’s instructions. Twenty-four h post-transfection, the media was changed to fresh media containing PTC124, and 48 h post-transfection the cells were collected and analysed using a BD Accuri C6 Flow Cytometer (BD Biosciences, Le Pont de Claix, France).

### Western blot analyses and *in vitro* prenylation assays

For the western blot analyses, cells were scraped into cold PBS containing protease inhibitors, resuspended in Laemmli’s sample buffer and analysed as previously described^29^. Similarly for the *in vitro* prenylation assays, pelleted cells were resuspended in degased prenylation lysis buffer and the unprenylated cytosolic Rab pool was analysed as described^29^. Quantifications were performed using the Image J software and expressed as a function of the β-actin signal.

### Statistical analyses

For the comparison of two groups of data, a Mann and Whitney test was used. For the comparison of three or more groups, a Kruskal-Wallis ANOVA was performed followed by a *post-hoc* 2 × 2 comparison^52^.

### *In silico* predictions and 3D modelling

The predictive effect of putative amino acid change on REP1 function was analysed using the online prediction programs PolyPhen-2 HumVar, SIFT, SNAP, PMut, MutationAssessor.org and PROVEAN software. The *CHM* and REP1 reference sequences used were RAE1_HUMAN, NP_000381 and ENSP00000350386. The 3D structure used to perform the analysis was a homology model derived from the Protein Data Bank (http://www.rcsb.org/) structure 1vg0_A (raw data kindly provided by Dr. Sergeev, NIH, Bethesda, USA) that was previously reported^53^. Pictures were drawn using the PyMOL Molecular Graphics System, (Version 1.8 Schrödinger, LLC, New York, USA).

### Data Availability Statement

All data generated or analysed during this study are included in this published article (and its Supplementary Information file).

## Electronic supplementary material

Supplementary information
