# Supplementary material for: The effect of PTC124 on choroideremia fibroblasts and iPSC-derived RPE raises considerations for therapy
Source: Sci Rep. 2018 May 29;8:8234. doi: 10.1038/s41598-018-26481-7 (PMC5974348; doi:10.1038/s41598-018-26481-7)
Supplement: Supplementary file 1 — Supplementary information [file 41598_2018_26481_MOESM1_ESM.pdf]

SUPPLEMENTARY INFORMATION

The effect of PTC124 on choroideremia fibroblasts and iPSC-derived RPE raises considerations for therapy

Simona Torriano, Nejla Erkilic, David Baux, Nicolas Cereso, Valerie De Luca, Isabelle Meunier, Mariya Moosajee, Anne-Francoise Roux, Christian P. Hamel and Vasiliki Kalatzis

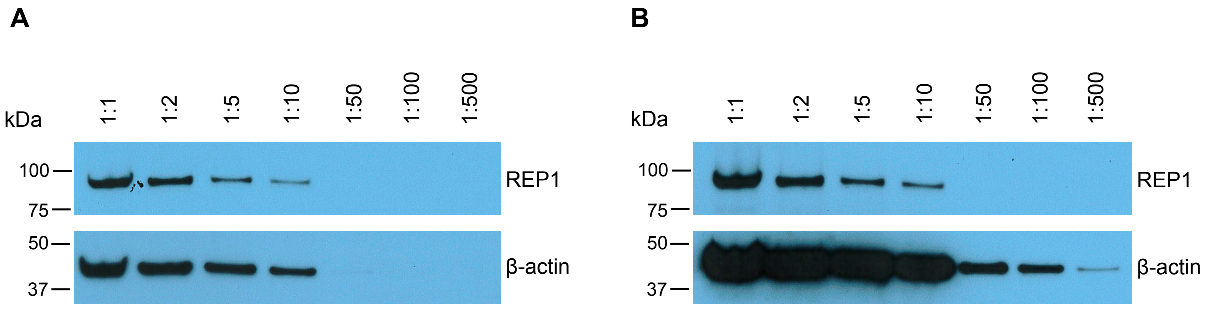

Supplementary Figure 1: Western blot analysis to determine the detection threshold of wild-type REP1 expression. Serial dilutions (1:2, 1:5, 1:10, 1:50, 1:100 and 1:500) of wild-type fibroblast cell lysates were analysed by hybridisation with a specific anti-REP1 antibody at short (**A**) and long (**B**) exposure times. REP1 expression was detectable up to a 1:10 dilution. β-actin serves as a loading control.

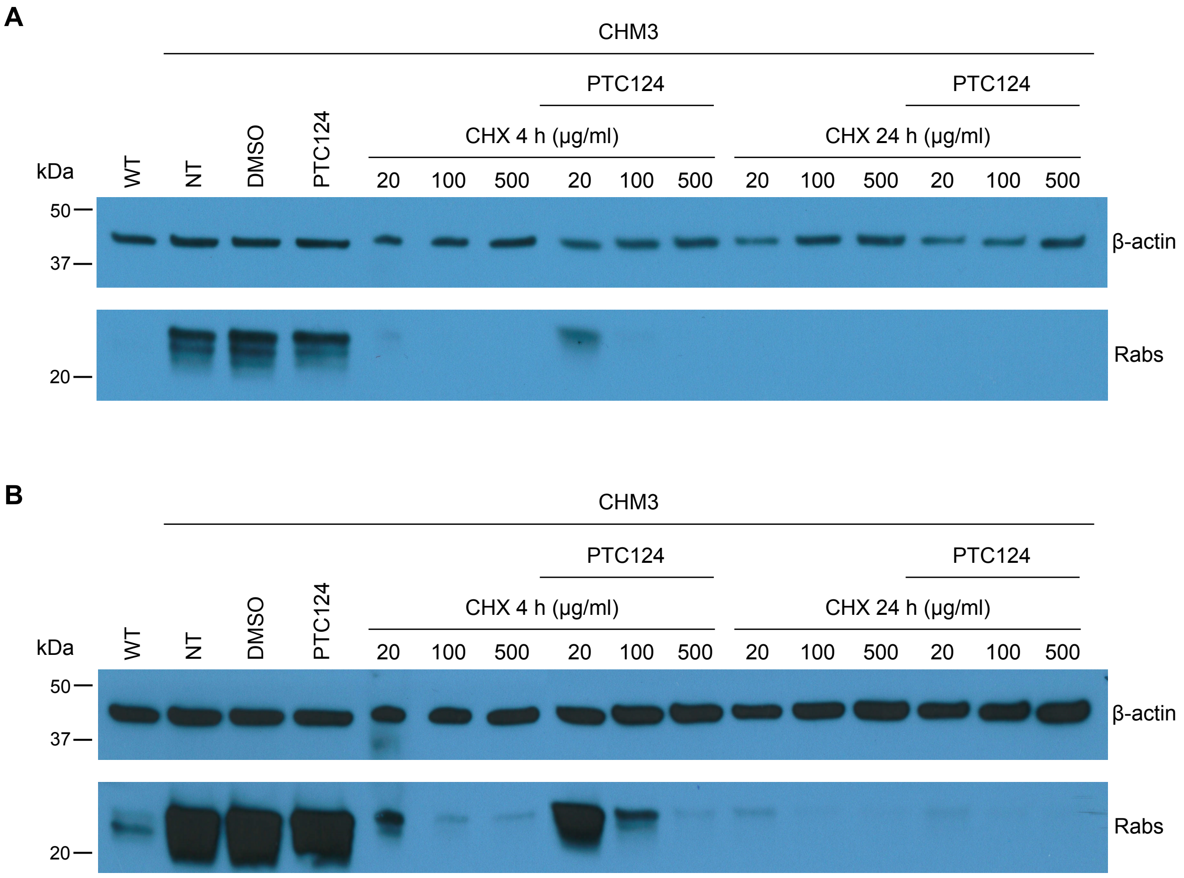

Supplementary Figure 2: Effect of lower CHX doses and shorter incubation time on the Rab pool in CHM3 fibroblasts. A) *In vitro* prenylation assay showing biotinylated Rab proteins in wild-type (WT), and in non-treated (NT), DMSO-treated or PTC124-treated CHM3 cells. β-actin serves as a loading control. **B**) Longer exposure time of the western blot shown in (A). Non-treated (NT), DMSO-treated and PTC124-treated CHM3 cells show high levels of biotinylated Rabs. Treatment with CHX reduced this Rab pool in a dose-dependent manner after 4 h incubation. The decrease in biotinylated Rabs was more pronounced after 24 h of CHX treatment. This effect was independent of PTC124.

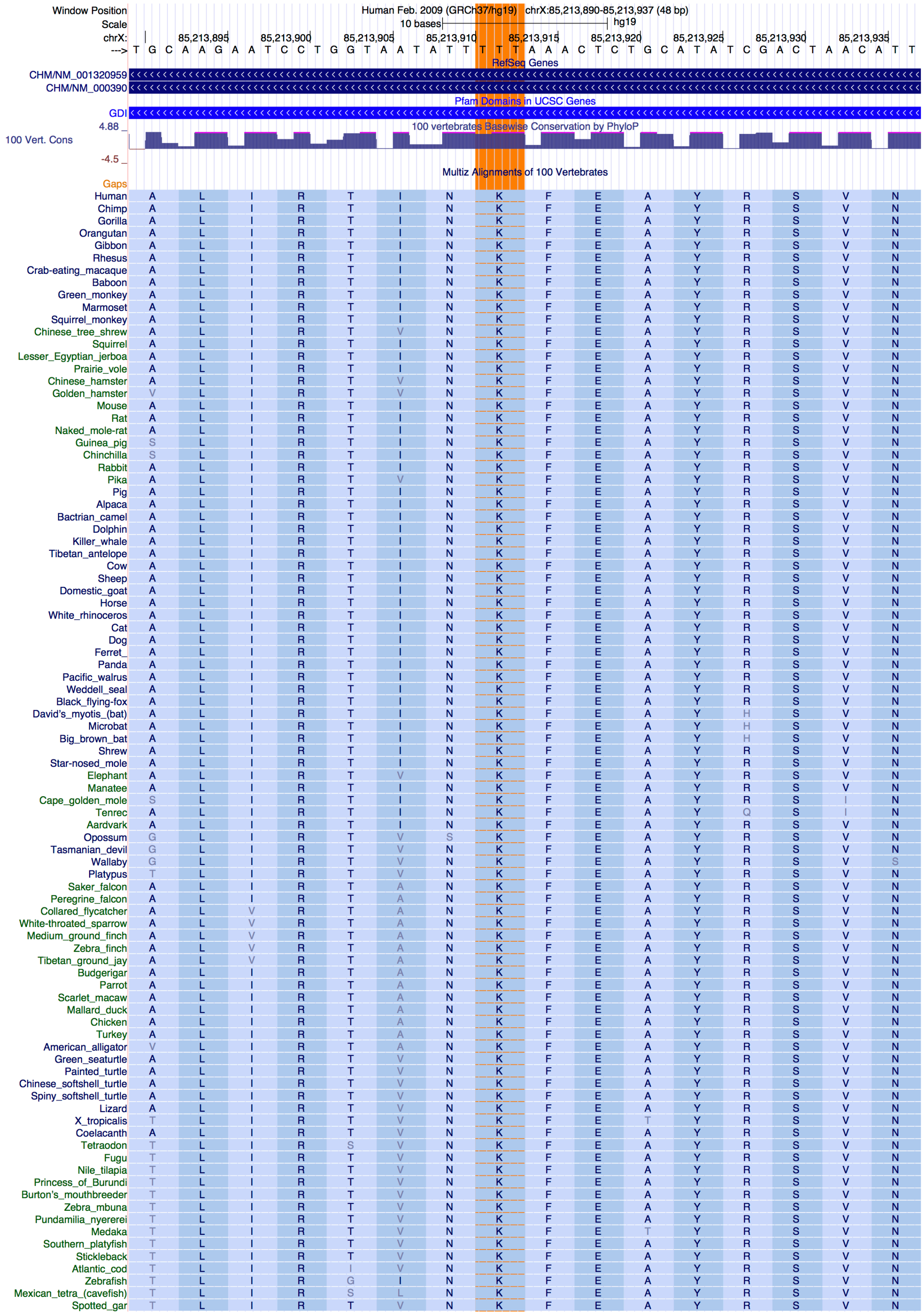

Supplementary Figure 3: Conservation of the K258 amino acid residue of human REP1. Sequence alignment showing the conservation of the lysine residue at position 258 (in orange) of human REP1 among 91 vertebrates species. Nine species with undefined sequences have been hidden. Screen shot obtained from the UCSC genome browser, http://genome.ucsc.edu.
